# Experiences of stigma, discrimination and violence and their impact on the mental health of health care workers during the COVID-19 pandemic

**DOI:** 10.1038/s41598-024-59700-5

**Published:** 2024-05-08

**Authors:** Miroslava Janoušková, Jaroslav Pekara, Matěj Kučera, Pavla Brennan Kearns, Jana Šeblová, Katrin Wolfová, Marie Kuklová, Dominika Šeblová

**Affiliations:** 1 Department of Epidemiology, Second Faculty of Medicine, Charles University, Prague, Czechia; 2 Division of Medical Psychology, Third Faculty of Medicine, Charles University, Prague, Czechia; 3 Medical College, Prague, Czechia; 4 National Institute of Mental Health, Klecany, Czechia; 5 Amsterdam Public Health Research Institute, Faculty of Science, Vrije Universiteit Amsterdam, Amsterdam, The Netherlands; 6 Paediatric Emergency Department, Motol University Hospital, Prague, Czechia; 7 Faculty of Science, Charles University, Prague, Czechia

**Keywords:** Psychological distress, Depression, Stigmatization, Violence, Health care workers, Mental health, Psychology, Health care, Health occupations, Risk factors

## Abstract

Health care workers have been exposed to COVID-19 more than people in other professions, which may have led to stigmatization, discrimination, and violence toward them, possibly impacting their mental health. We investigated (1) factors associated with stigma, discrimination, and violence, (2) the association of stigma, discrimination, and violence with mental health, (3) everyday experiences of stigmatization, discrimination, and violence. We chose a combination of a quantitative approach and qualitative content analysis to analyze data collected at three time points: in 2020, 2021 and 2022. A higher age was associated with lower odds of experiencing stigma, discrimination, and violence, whereas female gender was related to more negative experiences. The intensity of exposure to COVID-19 was associated with greater experience with stigmatization, discrimination, and violence across all three years (for example in 2022: odds ratio, 95% confidence interval: 1.74, 1.18–2.55 for mild exposure; 2.82, 1.95–4.09 for moderate exposure; and 5.74, 3.55–9.26 for severe exposure, when compared to no exposure). Stigma, discrimination, and violence were most strongly associated with psychological distress in 2020 (odds ratio = 2.97, 95% confidence interval 2.27–3.88) and with depressive symptoms in 2021 (odds ratio = 2.78, 95% confidence interval 2.12–3.64). Attention should be given to the destigmatization of contagious diseases and the prevention of discrimination, violence, and mental health problems, both within workplaces and among the public.

## Introduction

As a novel highly infectious disease, COVID-19 impacts all spheres of our everyday lives. Apart from direct consequences, the pandemic has also led to negative social effects, such as misinformation, fear and hatred in the public sphere, which can give rise to the stigmatization of people who are or might be infected^1^. Stigmatization is a process that starts with labelling a person with unfavorable characteristics and continues with negative emotional reactions (stereotypes). This leads to a separation of “us” and “them”, status loss, social exclusion, discrimination, bullying or even violence^2^. Discrimination is a result of prejudice leading to the suppression and loss of opportunities^3^.

COVID-19 triggered double risk of stigma for health care workers (HCWs). First, many of them were infected by COVID-19, facing *experienced (enacted) stigma*^4^. *Experienced (enacted) stigma* refers to experiences of stereotypes, prejudice and discrimination from others due to particular health condition^5,6^. Second, HCWs are associated with this disease through their work, so they are at high risk of *stigma-by-association*^7^. This type of stigma, also called *courtesy stigma*^8^, affects carers or close persons of people with stigmatized attributes. Moreover, HCWs can internalize negative attitudes, including shame, and apply such stereotypes to themselves and experience *self-stigmatization (internalized stigma)*^9^.

Stigmatization, discrimination and violence are serious negative issues that have emphasized the vulnerability of health care workers. Stigma due to COVID-19 can manifest in various areas of HCW’s life. It affects social relationships and communication within families^10^. This could be further observed as social avoidance^11^ or isolation or rejection^12,13^. The COVID-19 pandemic exacerbated verbal and physical violence against HCWs^14,15^. HCWs significantly experienced more COVID-19-related bullying than did those who worked in other settings^16^. In particular, HCWs reported conflicts with patients^17^ and COVID-19-related bullying and harassment from colleagues, authorities, neighbors, or the public^16,18^. Discrimination was also directed towards family members of HCWs; for example, they reported that their children were not invited to their friends’ homes or accepted to free-time activities^12^.

Various individual characteristics such as age, gender, occupational or educational level can be associated with experienced stigma, discrimination or violence at the workplace. . For example, in a study from Kashmir^19^, experienced stigma was significantly greater in men than in women; experienced stigma and internalized stigma were associated with high education and occupation level. Another study from India^20^ found out that age over 30 years, being a man, lower education, and being married were significantly associated with greater experienced stigma. Focusing on the age of staff members, younger nurses and men are generally at greater risk of experiencing aggression^21^.

Fighting stigma is necessary, as stigma can lead to serious health consequences^22^. Experiences of stigma and discrimination affect the mental health of stigmatized people. In particular, stigmatized people are at high risk of anxiety, depression, sleep problems^17,18^ and psychological distress^23,24^. COVID-19-related stigma is also associated with negative work outcomes, such as fatigue, burnout or dissatisfaction^25^. COVID-19 stigma is a new condition that affects the life of HCWs. However, there is a gap in the literature regarding the development of COVID-19-related stigma since the beginning of the pandemic and its changes over time, together with its effects on mental health. Therefore, we aimed to investigate (1) what factors are associated with experiencing stigma, discrimination, and violence among HCWs; (2) the association of experiencing stigma, discrimination, and violence with HCWs’ mental health problems; and (3) the content of everyday experiences of stigmatization, discrimination, and violence due to COVID-19 among HCWs. For this reason, we chose a quantitative approach followed by a qualitative research design to answer our research aims.

## Method

### Participants and study design

This study included participants enrolled in the Czech arm of the international COVID-19 Health caRe wOrkErS (HEROES) study. HEROES is a global prospective cohort study aiming to evaluate the impact of the COVID-19 pandemic on health care workers^26^. In the Czech Republic, data was collected at three time points: year 2020, 2021 and 2022. Baseline questionnaires were distributed in summer 2020 (24th of June to 30th of August—several weeks after the first state of emergency in Czechia), the first follow-up occurred in spring 2021 (15th of February to 31st of April—during the peak of the pandemic and a lock-down), and the second follow-up occurred in fall 2022 (15th September 2022 to 15th November 2022—after the end of pandemic measures). Workers in health care services (e.g., physicians, nurses, paramedics, nonmedical personnel) or social services were eligible for enrollment in the study without age limitations. We reached this population through a two-stage process. First, invitations to the study were distributed to health care facilities cataloged by the Ministry of Health, scientific societies, professional bodies and associations. Second, these organizations were asked to distribute the link to the questionnaire to their members or employees and confirm the distribution to our study team. There were 1,778 respondents in year 2020, 1,840 in year 2021, and 1,451 in year 2022. Some of them took part in the study at two or three years, and some took part in only one year. In the present study, we analyzed the data as three repeated cross-sectional surveys, investigating the associations per year separately. We excluded individuals with missing data on basic socio demographic measures, resulting in 1,731 individuals in year 2020, 1,809 in year 2021, and 1,398 in year 2022.

All participants provided informed consent prior to responding to the online survey. The HEROES study was approved by the Columbia University Institutional Review Board. The Czech arm of the HEROES Study was approved by the Ethics Committee of the Ministry of Health as well as the Ethical Review Board of the University Hospital Motol, Prague, Czech Republic. All methods were performed in accordance with relevant guidelines and regulations.

### Measures

#### Experience of stigmatization, discrimination, or violence

The information on the negative experience of stigmatization, discrimination, or violence (further negative experiences) is derived from two statements: (1) I have felt stigmatized or discriminated against as a health worker due to the COVID-19 pandemic. (2) I have experienced violence due to being a health worker during the pandemic. Possible answers were “strongly disagree”, “disagree”, “agree” and “strongly agree”, and were rated on a scale from 1 to 4. We considered “agree” or “strongly agree” (3 or 4 points) to indicate experienced stigmatization, discrimination, or violence, respectively. In the descriptive analysis, we separately presented the frequency of experienced stigmatization or discrimination (yes vs. no) and experienced violence (yes vs. no). Given that there was a relatively low number of participants with experiences of violence, for multivariable analysis, we combined the two answers, constructing one binary variable (stigmatization, discrimination, or violence: yes vs. no), as follows: Participants who reached 3 or 4 points in at least one of the two statements were considered to be experiencing stigmatization, discrimination, or violence.

#### Exposure to COVID-19

Data on exposure to COVID-19 were acquired from four variables defining four different conditions describing proximity to the illness: (1) contact with patients with COVID-19, captured if participants were in close contact with patients with suspected or confirmed COVID-19 disease during the past week); (2) experience of death due to COVID-19 in someone close to them, which was created from 3 items: if their patient was at work or someone close to them died from COVID-19 since the beginning of the pandemic; (3) prioritization of patients, captured if they had to decide how to determine the priority of individual patients with COVID-19; and 4) the COVID-19 unit, captured if they worked at a specific COVID-19 unit (available only in wave 1 and wave 2). Based on these variables, we created four levels of exposure to COVID-19: none, mild (one condition), moderate (two conditions), and severe (three or four conditions).

#### Distress

The level of distress was measured by the validated version of the 12-item General Health Questionnaire (GHQ-12), which is suitable for the assessment of psychological distress in nonclinical samples^27^. The instrument detects short-term changes in mental health and in levels of psychological functioning. Respondents are asked if they have recently experienced a particular symptom or behavior. Each item is rated on a four-point scale (less than usual, no more than usual, more than usual, or much more than usual). We used a Likert scoring system (0–1–2–3), for a maximum possible score of 36 points. We created a binary variable, psychological distress, using a cut-off score of ≥ 15 points, which is based on previously published recommendations^28^.

#### Depressive symptoms

Depressive symptoms were evaluated by the 9-item Czech version of the Patient Health Questionnaire (PHQ-9), which measures the severity of depression. Respondents were asked how often during the past two weeks they experienced the symptom, with possible response options of “not at all”, “several days”, “more than half the days” and “nearly every day” (scoring 0–1–2–3), for a total possible score of 27 points. We created a binary variable on depressive symptoms, using a cut-off score ≥ 5 points, corresponding to a greater risk of mild to severe depression, which is based on a study by Kroenke et al.^29^.

#### Other participants’ characteristics

We considered the following characteristics of the respondents: age (years), gender (man vs. woman), occupation (physician, nurse, management or other) and living alone (yes vs. no). With respect to gender, the participants had three options how to characterize themselves: man, woman or other, which reflects the non-binary construct of gender. In our study sample, no participant chose the option “other”. In this article, given that the question was non-binary, we refer to gender, rather than sex. Concerning occupation, paramedics, laboratory technicians, technical staff, administrative workers and IT staff were among our respondents characterized as “other” occupations. Some of them did not experience the direct contact with COVID-19 patients but were also affected by the pandemic situation.

#### Qualitative measure

In all years, data from an open-ended question on general experiences regarding the COVID-19 pandemic were analyzed: “Is there anything we did not ask that you would like to add, so we can better understand the experiences of workers like you during this pandemic?”. In the case of year 2022, one question was added for a better understanding of the impacts of long-term COVID-19 on the mental well-being of HCWs: “How has COVID-19 affected your mental well-being in various areas of your life?”, which was also analyzed.

#### Data analysis

We performed the quantitative analysis in several steps. First, we summarized the descriptive characteristics of the participants at each year as the frequency (n, %) and mean ± standard deviation (SD). Differences between waves were assessed using analysis of variance (ANOVA) or chi-squared test were appropriate. Second, we investigated the association between participants’ characteristics and their experience of stigmatization, discrimination, or violence in each wave. We employed logistic regression to estimate the odds ratio (OR) with 95%confidence interval (CI) for the association of participant characteristics (Model 1: age, gender, occupation and living alone; Model 2: added also exposure to COVID-19) with the experience of stigmatization, discrimination, or violence. Next, we investigated the association of the experience of stigmatization, discrimination, or violence with mental health outcomes. We used logistic regression to estimate ORs with 95%CIs for the association of the experience of stigmatization, discrimination, or violence with distress, adjusting for age, gender, occupation and living alone. In the end, we repeated the previous step by investigating depressive symptoms as the outcome instead of distress. Missing data were imputed using the multiple imputation with chained equations (MICE) algorithm, resulting in 10 imputed datasets^30^. MICE is a robust and informative method that imputes data using an iterative series of predictive models. In our analysis, characteristics other than participants' age were imputed. The imputed datasets were analyzed separately, after which the results were pooled using Rubin’s rules. As a sensitivity analysis, we checked the robustness of our findings by repeating the analysis using only complete cases. Given that we found similar and consistent results to the analysis using the sample in which missing data were imputed, we do not present the results of this sensitivity analysis in this paper. Sensitivity analysis for separated variables (Experiences with stigma/discrimination and Experiences with violence) could be found in Supplemental Tables S1 and S2. In Supplementary Table S3 participant characteristics stratified by number of wave presence are presented. The analyses were performed in R version 4.2.2.

Open-ended data were analyzed using the conventional approach to qualitative content analysis^31^, which is suitable for obtaining a descriptive understanding of particular issues^32^. We removed unfilled, unclear or incomplete responses from the analysis. Furthermore, one author (BLIND) evaluated all the written responses and created an initial coding scheme that was consulted with two other authors (BLIND). To minimize bias, we used multiple coding approaches^33^. Two researchers (BLIND) independently coded five random pages from each subset of open-ended questions according to this initial coding framework and then compared their findings with those of the first author (BLIND) and established a final coding scheme that was used to code all the data in ATLAS.ti Version 7.5. Codes were then sorted into categories, which were used to identify underlying meanings and themes, as commonly performed in content analysis^34^. Since the open-ended questions in the research project were rather general, only answers related to the themes “stigmatization”, “discrimination”, and “violence” were used for the purpose of this study. Themes were presented as the following categories: stigma, self-stigma, discrimination, and violence. In particular, we included answers from 244 participants (from 1969 total valid answers after removing missing data): particularly 62 in 2020, 61 in 2021, 121 in 2022.

### Ethics approval

Ethics approval was obtained from the Ethics Committee of the Czech Ministry of Health (MZDR-23393/2020–1/MIN/KAN) and Ethics Committee at the 2nd Faculty of Medicine (EK-753.3.6121).

## Results

### Characteristics of participants

We studied 1,731 HCWs at year 2020 (average age 44 years, 77% women), 1,809 at year 2021 (46 years, 75% women), and 1,398 at year 2022 (46 years, 75% women); the descriptive characteristics of the HCWs are presented in Table 1. The experience of stigmatization or discrimination showed a slight declining trend, with 30% of respondents reporting it at year 2020, 26% at year 2021, and 25% at year 2022. Such a trend did not appear for the experience of violence, as this occurred in 5% of the participants at years 2020 and 2021, but in 12% at year 2022. Mental health problems had the lowest frequency at year 2020, with a disproportionately greater frequency at year 2021, which corresponded to peak COVID-19 rates in the Czech Republic, and then again less frequent at year 2022. However, the prevalence of mental health problems at year 2022 did not reach the lowest levels present at year 2020. Specifically, distress was found in 22% of the respondents at year 2020, 48% at year 2021, and 25% at year 2022. Depressive symptoms occurred in 37% of the HCWs at year 2020, 56% at year 2021, and 43% at year 2022. All observed changes were found to be statistically significant.

**Table 1:** Participant characteristics.

Variable	Year 2020	Year 2021	Year 2022
	(N = 1731)	(N = 1809)	(N = 1398)
Experience of stigmatization, or discrimination (n, %)	515 (29.8%)	471 (26.0%)	344 (24.6%)
Experience of violence (n, %)	85 (4.9%)	85 (4.7%)	165 (11.8%)
Experience of stigmatization, discrimination, or violence (n, %)	553 (31.9%)	507 (28.0%)	396 (28.3%)
Psychological distress, n (%)	386 (22.3%)	859 (47.5%)	354 (25.3%)
Mild to severe depressive symptoms, n (%)	636 (36.7%)	1019 (56.3%)	605 (43.3%)
Exposure to COVID-19, n (%)			
None	1159 (67.0%)	406 (22.4%)	403 (28.8%)
Mild	362 (20.9%)	509 (28.1%)	455 (32.5%)
Moderate	118 (6.8%)	570 (31.5%)	381 (27.3%)
Severe	92 (5.3%)	324 (17.9%)	159 (11.4%)
Age, mean ± SD	44.1 (11.9)	45.7 (12.2)	45.8 (11.3)
Women, n (%)	1325 (76.5%)	1361 (75.2%)	1042 (74.5%)
Occupation, n (%)			
Physician	484 (28.0%)	738 (40.8%)	390 (27.9%)
Nurses	754 (43.6%)	673 (37.2%)	606 (43.3%)
Management	263 (15.2%)	217 (12.0%)	229 (16.4%)
Other	230 (13.3%)	181 (10.0%)	173 (12.4%)
Living alone, n (%)	213 (12.3%)	268 (14.8%)	220 (15.7%)

*SD* standard deviation; Differences between waves were assessed using analysis of variance (ANOVA) or chi-squared test.

### Factors associated with negative experiences of stigmatization, discrimination and violence

Table 2 presents the associations of participants’ characteristics with their negative experiences. A higher age was slightly but consistently associated with lower odds of experiencing stigmatization, discrimination, or violence across waves. Being a woman was related to more experience of stigmatization, discrimination, or violence, but these associations differed across years and models. In Model 1, when only sociodemographic characteristics were entered into the model, the association was present only at year 2021, and women had 33% greater odds of reporting stigmatization, discrimination, or violence (OR 1.33; 95%CI 1.03–1.71) than men did. In Model 2, when participants were also exposed to COVID-19, being a woman was more strongly associated with her negative experiences at both year 2021 (OR 1.50; 95%CI 1.15–1.95) and year 2022 (OR 1.43; 95%CI 1.01–2.04). Compared to physicians, nurses or managerial staff did not show different odds of experiencing stigmatization, discrimination, or violence. According to Model 1, staff who did not belong to any of these groups were less likely to report experiencing stigmatization, discrimination, or violence at year 2020 (OR 0.57; 95%CI 0.39–0.84) or year 2021 (OR 0.49; 95%CI 0.31–0.79). When exposure to COVID-19 was considered, the association persisted only at year 2020 (OR 0.66; 95%CI 0.44–0.98). Living alone was not associated with these negative experiences.

**Table 2:** Association of participants’ characteristics with experience with stigmatization, discrimination, or violence.

	Experience with stigmatization, discrimination, or violence		
	Year 2020	Year 2021	Year 2022)
Variable	OR [95%CI]		
Model 1			
Age	**0.98 (0.97–0.99)**	**0.97 (0.96–0.98)**	**0.97 (0.96–0.99)**
Gender			
Man	Ref.		
Woman	1.07 (0.82–1.39)	**1.33 (1.03–1.71)**	1.23 (0.89–1.71)
Occupation			
Physician	Ref.		
Nurse	0.94 (0.72–1.23)	0.85 (0.66–1.09)	0.98 (0.69–1.39)
Management	0.8 (0.56–1.16)	0.75 (0.49–1.15)	0.89 (0.58–1.37)
Other	**0.57 (0.39–0.84)**	**0.49 (0.31–0.79)**	0.63 (0.33–1.22)
Living alone			
No	Ref.		
Yes	1.22 (0.86–1.73)	0.88 (0.62–1.26)	1.23 (0.84–1.82)
Model 2			
Exposure to COVID-19			
None	Ref.		
Mild	**2.01 (1.53–2.64)**	**1.77 (1.22–2.57)**	**1.74 (1.18–2.55)**
Moderate	**2.76 (1.73–4.4)**	**2.67 (1.88–3.8)**	**2.82 (1.95–4.09)**
Severe	**2.99 (1.84–4.86)**	**3.87 (2.61–5.74)**	**5.74 (3.55–9.26)**
Age	0.99 (0.97–1.00)	**0.98 (0.97–0.99)**	**0.97 (0.96–0.99)**
Gender			
Man	Ref.		
Woman	1.18 (0.89–1.55)	**1.50 (1.15–1.95)**	**1.43 (1.01–2.04)**
Occupation			
Physician	Ref.		
Nurse	0.95 (0.73–1.25)	0.95 (0.74–1.23)	1.06 (0.74–1.53)
Management	0.81 (0.56–1.16)	0.89 (0.57–1.38)	1.05 (0.68–1.63)
Other	**0.66 (0.44–0.98)**	0.79 (0.48–1.29)	1.05 (0.52–2.13)
Living alone			
No	Ref.		
Yes	1.16 (0.81–1.65)	0.85 (0.6–1.22)	1.22 (0.81–1.82)

*OR* Odds ratio, *CI* Confidence interval, Significant results (*p* < 0.05) are in bold.

Mild, moderate, and severe exposure to COVID-19 had a graded association with negative experiences when compared to no exposure, indicating that each level was associated with increased risk. With regard to trends over time, the association between mild exposure and these negative experiences was strongest at year 2020 (OR 2.01; 95%CI 1.53–2.64) and weaker in later years (year 2021: OR 1.77; 95%CI 1.22–2.57; year 2022: OR 1.74; 95%CI 1.18–2.55). In contrast, the association between severe exposure and these negative experiences showed an opposite increasing pattern across the waves (year 2020: OR 2.99; 95%CI 1.84–4.86; year 2021: OR 3.87; 95%CI 2.61–5.74; year 2022: OR 5.74; 95%CI 3.55–9.26). There was no evident pattern for moderate exposure.

### Association of negative experiences with mental health problems

The negative experiences were related to both distress and depressive symptoms at all years (Table 3). The association between these negative experiences and distress was strongest at year 2020 (OR 2.97; 95%CI 2.27–3.88) and then gradually decreased (year 2021: OR 2.50; 95%CI 1.99–3.15); year 2022: OR 1.54; 95%CI 1.13–2.08). Such a trend was not present for the association with depressive symptoms (year 2020: OR 2.44; 95%CI 1.90–3.12; year 2021: OR 2.78; 95%CI 2.12–3.64; year 2022: OR 1.63; 95%CI 1.25–2.12).

**Table 3:** Associations of experience of stigmatization, discrimination, or violence with distress and depression across years.

Year	Distress	Depressive symptoms
	OR [95%CI]	OR [95%CI]
2020	2.97 (2.27–3.88)	2.44 (1.90–3.12)
2021	2.50 (1.99–3.15)	2.78 (2.12–3.64)
2022	1.54 (1.13–2.08)	1.63 (1.25–2.12)

*OR* Odds ratio, *CI* Confidence interval. Adjusted for age, gender, occupation and living alone.

### Sensitivity analysis

Results of sensitivity analysis in Supplementary Table S1 largely followed the main analyses, but many estimates were imprecise. Several differences in results are worth noting: the odds for exposure to COVID-19 were higher among those with negative experiences with violence and all categories of occupation (nurses, management, other) had higher odds of experiencing stigma/discrimination or violence compared to physicians. The sensitivity analysis presented in Supplementary Table S2 indicates that the dynamic of association between the both types of negative experiences (stigma/discrimination and violence) and mental health problems followed a similar direction. However, experiences of violence had slightly lower odds of depressive symptoms than the experiences of stigma and discrimination. With respect to the experience of stigmatization/violence, difference between those that participated only in one wave and those in at least two waves, the two groups were comparable, except for violence experience separately. Those present in only one wave had slightly higher frequency of experienced violence, distress and depression (see Supplementary Table S3).

### Manifestations of stigmatization, discrimination and violence

We identified four main categories: stigmatization, self-stigmatization, discrimination, and violence. Table 4 presents their particular manifestations (subcategories) and exemplar quotations. Many HCWs expressed experiences of stigmatization by the public and media, especially at year 2022. Many of them described situations of avoidance by colleagues (often co-workers from their own department, managers, and health care professionals from other departments or specialties) and close persons and defamation at work. One specific manifestation was ridicule which was mentioned by several participants. HCWs described discriminatory behavior most often at work. In particular, they experienced unfair financial remuneration, violations of working conditions and bossing. Some of HCWs experienced discriminatory work-life balance conditions. In 2022, discrimination was directed against unvaccinated people, as many participants described. Self-stigmatization refers to the internalization of negative attitudes and prejudice. A few HCWs separated themselves from their families due to excessive fear of the infection at the beginning of the pandemic. In further waves, some felt remorse and self-blamed themselves for the possible infection and death of a person in their neighborhood. Violence was depicted by many HCWs at the level of verbal aggression from patients and their families, the public and the community (no one mentioned a case of physical violence). Experience with verbal aggression was minimal at the beginning of the pandemic and increased over time.

**Table 4:** Outcomes of qualitative content analysis.

Categories and subcategories	Example quote
Stigmatization	
Avoidance by colleagues and family members	“I was afraid to go to the communal toilet at work, even if I only had a cold. My colleagues looked at me like a murderer who is selfishly spreading a deadly contagion”. (2022)
Public prejudice	“The problem was the public's interest and gratitude—during the first wave of the pandemic we were considered heroes, once the situation calmed down, we were considered incompetent, heartless and greedy again”. (2020)
Defamation at work	“Ignorance from the hospital management and supervisors, misinformation, dehumanization of the hard work everyone does there. No sign of respect, it is very humiliating”. (2022)
Ridicule	“Currently, wearing a face mask in public often provokes ridicule from passers-by”. (2020)
Discrimination	
Violation of the Labor Code and rules, bossing	“In one month, I even had to agree to eleven 24 h shifts a month. If I refused to do more work, the managers were unpleasant and threatened me”. (2022)
Remuneration	“We're working in a lab environment that investigates Covid. As health care workers, we were not more protected or rewarded for risk as direct health workers”. (2021)
Non-vaccination	“There was a lot of pressure (ordering) to get vaccinated at our workplace. When a person refused, he was accused of treason”. (2022)
Personal life	“Some parents did not want the children of the HCWs to go to class with their children. I had to continue educating my own children at home, which was very difficult to combine with working in a hospital”. (2020)
Self-stigmatization	
Self-guilt and remorse	“The constant fear of infecting loved ones—children, grandchildren, parents—the fear of seeing family and the guilt of meeting them”. (2020)
Separation	“I was worried about myself, but even more worried about infecting my wife if I got sick. The concern was so strong that I moved into a separate sublet for three months”. (2020)
Verbal violence	
Patients and their families	“Every day we face verbal aggression from patients, nervousness, especially towards female physicians”. (2022)
Public, community	“Overall, since the pandemic (and probably also in connection with the war in Ukraine), I perceive a greater polarization of society, conflicts in families due to different opinions and increasing aggression”. (2022)

Identification of participants—year of the survey.

## Discussion

In the present study, we investigated how HCWs in the Czech Republic experienced stigmatization, discrimination, and violence during the COVID-19 pandemic. HCWs are particularly vulnerable to experiencing stigmatization and discrimination due to their exposure to patients suspected of being infected. The experience of stigmatization and discrimination was reported as the highest at the first data collection and then slightly decreased, whereas the experience of violence was reported as the highest in the latest data collection. The intensity of exposure to COVID-19 was associated with stigmatization, discrimination, and violence. HCWs with these negative experiences had greater odds of experiencing both psychological distress and depressive symptoms. However, experiences of violence had slightly lower odds of depressive symptoms than the experiences of stigma and discrimination. According to qualitative analysis, HCWs experienced stigmatization often by the public, media, colleagues, and managers. Discriminatory behavior was connected with work conditions, remuneration, and refusal to get vaccinated in the latest wave. Violence manifested as verbal aggression from patients and their family members, the public and the community.

This study provides novel information about the development of a recently emerged stigmatized condition. We had the opportunity to explore its development from the beginning—the first survey took place 4–5 months after the beginning of the pandemic in the Czech Republic—until the late stage in 2022. Our results showed that increased exposure to patients with COVID-19 is related to increased experience with stigmatization, discrimination, or violence. Approximately one-quarter of the HCWs experienced stigmatization and discrimination, which corresponds to lower levels than those reported in three meta-analyses^4,17,35^. The experience of stigmatization may diminish over time as people learn to cope and become more resilient and build self-esteem and self-efficacy^36^. Accordingly, our results showed that the endorsement of discrimination and stigmatization was highest in 2020 and slightly decreased in later data collections. The decline could be influenced by mass media interventions^37^ aimed at the general public to reduce stigma in the immediate, short and medium term, such as campaigns to correct myths, rumors and stereotypes and to challenge prejudice^38^ in the form of stories and conditions to cultivate empathy and social change, as reported in recent strategies^7^.

In contrast, violence could be a consequence of stigmatization and negative public attitudes that develop over time and manifest later as frustration increases. Although the occurrence of workplace violence among Czech HCWs is lower than that reported in a previous meta-analysis^39^, the trend in the data is the same. This tendency could be explained by an increased number of patients and their long-term stress and dissatisfaction caused by the pandemic and by an urge to direct frustration toward HCWs^40^. The Czech people could also be frustrated by governmental restrictions and regulations and express their anger in the form of demonstrations and verbal violence. The lower occurrence of violence in our study could be explained by the fact that violence could also be included in answers to a question exploring experiences with stigmatization and discrimination. Our questionnaire inquired about a general experience with violence, but the outcomes of qualitative analysis showed that HCWs described only experiences with verbal violence.

Women in our sample reported experiencing stigmatization, discrimination, or violence more often than men did. This finding is in line with the results of reviews that revealed that women were more often stigmatized and discriminated against^4,41^. This finding could be explained by the fact that women historically constitute an oppressed group that holds less power and prestige than men^2,36^. However, not all related studies are consistent with these results; others have shown that perceived stigma is greater in men than in women^19,42^. It seems gender is not a clear discriminating factor; it may depend on the cultural and social context. In our study, we observed an association between being a woman and these negative experiences only in some yearsand in some models, suggesting that the association with gender is not consistent, changes with time and depends on contextual factors. Specifically, in our study, when only basic characteristics were adjusted for, women were more likely to report being victims of stigmatization, discrimination or violence only at the year 2021. However, the magnitude of the association to reporting these negative experiences increased, when proximal exposure to COVID-19 was included into the model. We speculate that the exposure to discrimination and violence in men may be explained by their actual exposure to COVID-19, while for women other dynamics may play a role. However, in our study, we were not able to disentangle these mechanisms into more detail.

In some studies, nurses were at higher risk of stigmatization and discrimination than other professionals were^43,44^, but our findings do not support this. We found that staff who were not physicians, nurses or management staff members had lower odds of experiencing stigmatization, discrimination, or violence in the early and middle waves, which corresponded to the years 2020–2021. In contrast, we did not detect any between other occupations and such experiences. Further, the experience of stigmatization, discrimination, or violence was related to younger age. This is in line with the fact that younger adults might embrace their use of social media and an increase in economic challenges facing younger people during this time, as well as the demands of childcare and schooling at home^45^. This is also the case for some studies claiming that younger HCWs are more vulnerable than their older colleagues because they have more experience, higher levels of self-confidence and greater resistance to stress^46,47^.

Our study showed that experiences of stigmatization, discrimination, or violence are consistently associated with an increased occurrence of mental health problems. However, we found that the associations of these negative experiences with distress and depressive symptoms may show distinct trends. Specifically, after the beginning of the pandemic (year 2020), the relationship with distress was greatest. However, as the pandemic progressed (year 2021), the magnitude of the association with distress slightly declined. In contrast, such a trend was not apparent for the association with depressive symptoms, where the highest risk was present in 2021, which corresponded to the highest COVID-19 spread. Thus, different mechanisms may be involved that could explain how the experience of stigmatization, discrimination, or violence relates to these distinct mental health problems. For example, the initial greater association with psychological distress could be a result of the unknown, new negative experiences and additional stress of hiding a stigmatized identity^48^. However, HCWs may have adapted to them and learned some coping strategies, which could result in lower distress at a later time. In contrast, we speculate that adaptation to these negative experiences may not influence depressive symptoms, which may lead to more severe mental health problems, which are not easy to adapt to. However, we acknowledge that our study was cross-sectional, and we cannot exclude reverse causality. It is also possible that people who are in distress or are depressed due to other causes more frequently report such negative experiences, which may be partially viewed subjectively.

Qualitative analysis described manifestations of COVID-19-related stigmatization, discrimination, and violence during the pandemic in the Czech Republic. In contrast to other findings^16,49^, we did not identify experiences with harassment by police or governmental officials. Instead, our participants often described experiences of stigma and violence by patients and patients’ relatives. Experiences of HCWs regarding avoidance by close persons, such as friends or family members could be described as an *apathetical stigma*^50^ relating to a lack of empathy toward family members, friends, or relatives when they are infected with the illness. *Self-stigmatization* was manifested by self-guilt and self-isolation, which is in line with the findings of a Finnish study that described stress to meet people and fear of being blamed^51^ and with a Japanese study^52^ that discovered self-imposed coping behavior based on feelings of guilt and keeping oneself isolated. Sources of both stigmatization and self-stigmatization could be uncertainty about the disease and fear of contagiousness^53^. Nevertheless, governmental measures of social distancing make the boundaries between social distance and stigma less apparent^12^.

### Strengths and limitations

Several limitations need to be mentioned. The respondents were not selected randomly. Therefore, this study may suffer from sampling bias, and the participants do not fully represent the population of health care workers in the Czech Republic. Nonresponse bias may also influence our results. A few studies suggest that worse mental health is associated with survey nonresponse^54,55^, however, other authors suggest that individuals may be more inclined to respond to mental health issues that concern them^56^. We also speculate that a significant portion of individuals experiencing distress may be less willing to participate due to their high workload commitments. Sensitivity analysis indicates that those respondents who took part in only one wave of survey experienced more violence, had greater COVID-19-exposure and more mental health problems when compared to those who responded in more waves. We suggest that the burden of mental health problems was underestimated in our study. It is possible that even the experience and reporting of stigmatization, discrimination, and violence, which may be associated with an individual's mental health^48^, may be underestimated. This may subsequently lead to the weakening and imprecision of the studied associations. Another drawback is that this study does not use an established measure of stigma, discrimination, or violence. The simplicity of the survey question prevents us from examining the complex nature of these constructs. In addition, the relatively small sample size prevents us from performing subgroup analyses, including those involving a greater variety of covariates and determining which factors may moderate the studied associations. In the end, we acknowledge that our study was focused on comparing data collected in three different years, however, the data collection was conducted in various months. We could not take into account a number of confounding factors related to the seasonality of COVID-19 as well dynamic fluctuations in mental health within a year. This study is unique due to its long-term perspective on stigmatization, discrimination and violence during the COVID-19 pandemic and benefits from a combination of qualitative and quantitative methodologies. It includes a robust sample that includes all regions of the Czech Republic, both university and regional hospitals, and various professions (doctors, nurses, managers, administrative and technical staff). The region of Central and Eastern Europe, to which the Czech Republic belongs, has been largely underrepresented in mental health research, despite the disproportionately greater burden of mental health problems on the European scale^57^. While the qualitative component is based on the results of a generally posed open-ended question, its outcomes provide a rich depiction of various manifestations of stigmatization, discrimination and violence that health care workers experienced during the COVID-19 pandemic.

## Conclusion

The results of this study suggest that HCWs are at risk of stigmatization, discrimination and violence that affect their mental health. This finding implies that attention should be given to the prevention of stigmatization of contagious diseases, discrimination, violence and mental health problems at the workplace and public levels. Interventions with a protective effect on the improved wellbeing of HCWs should be implemented at the workplace through the engagement of middle management^14^. All employees in health care should be provided with supervision and psychological help. Workplaces should also implement anti-violence strategies and violence prevention training^39^. The reduction of negative social aspects of the job in health care, such as a lack of support from management, psychological stress, excessive demands, long-term shortages of personnel, and job insecurity, should also be addressed.

### Supplementary Information

Supplementary Tables.

## Data Availability

Data are available on reasonable request from the senior author of this study.
