# Supplementary material for: Experiences of stigma, discrimination and violence and their impact on the mental health of health care workers during the COVID-19 pandemic
Source: Sci Rep. 2024 May 8;14:10534. doi: 10.1038/s41598-024-59700-5 (PMC11078939; doi:10.1038/s41598-024-59700-5)
Supplement: Supplementary file 1 — Supplementary Tables. [file 41598_2024_59700_MOESM1_ESM.docx]

**Supplement**

Table S1: Association of Participants´ Characteristics with Experience with Stigmatization or Discrimination, and Violence separately.

|  | Experience with stigmatization or discrimination | | | | Experience with Violence | | | |  |
| --- | --- | --- | --- | --- | --- | --- | --- | --- | --- |
|  | Year 2020 | Year 2021 | Year 2022 | Year 2020 | | Year 2021 | Year 2022 |  |  |
| Variable | OR [95% CI] | | | | | | | | |
| Model 1 |  |  |  |  | |  |  |  |  |
| Age | **0.98 (0.97 – 0.99)** | **0.97 (0.96 – 0.98)** | **0.98 (0.96 – 0.99)** | **0.97 (0.94 – 0.99)** | | **0.97 (0.95 – 0.99)** | **0.97 (0.95 – 0.99)** |  |  |
| Gender |  |  |  |  | |  |  |  |  |
| Man | Ref. |  |  |  | |  |  |  |  |
| Woman | 1.13 (0.86 – 1.5) | **1.34 (1.02 – 1.75)** | 1.36 (0.96 – 1.92) | 0.84 (0.47 – 1.5) | | 1.04 (0.61 – 1.78) | 0.94 (0.58 – 1.53) |  |  |
| Occupation |  |  |  |  | |  |  |  |  |
| Physician | Ref. |  |  |  | |  |  |  |  |
| Nurse | **1.63 (1.14 – 2.32)** | 1.64 (0.98 – 2.75) | 1.88 (0.98 – 3.61) | 1.2 (0.56 – 2.59) | | 3.16 (0.95 – 10.53) | 2.25 (0.99 – 5.11) |  |  |
| Management | **1.81 (1.23 – 2.67)** | **2.09 (1.22 – 3.6)** | 1.84 (0.95 – 3.56) | 0.87 (0.37 – 2.09) | | 2.11 (0.62 – 7.25) | 2.04 (0.84 – 4.96) |  |  |
| Other | 1.33 (0.85 – 2.08) | 1.47 (0.82 – 2.64) | 1.6 (0.73 – 3.48) | 2.01 (0.84 – 4.78) | | 3.37 (0.93 – 12.12) | 2.61 (1 – 6.83) |  |  |
| Living alone |  |  |  |  | |  |  |  |  |
| No | Ref. |  |  |  | |  |  |  |  |
| Yes | 1.29 (0.91 – 1.81) | 0.96 (0.66 – 1.4) | 1.2 (0.8 – 1.78) | 0.78 (0.33 – 1.81) | | 1.19 (0.61 – 2.31) | 1.59 (0.97 – 2.63) |  |  |
| Model 2 |  |  |  |  | |  |  |  |  |
| Exposure to COVID–19 |  |  |  |  | |  |  |  |  |
| None | Ref. |  |  |  | |  |  |  |  |
| Mild | **1.83 (1.38 – 2.43)** | **1.85 (1.27 – 2.69)** | 1.48 (0.99 – 2.22) | **2.38 (1.22 – 4.63)** | | 1.92 (0.84 – 4.39) | 1.72 (0.91 – 3.25) |  |  |
| Moderate | **2.58 (1.62 – 4.11)** | **2.87 (2 – 4.12)** | **2.42 (1.6 – 3.67)** | **2.7 (1.06 – 6.9)** | | 1.49 (0.66 – 3.4) | **3.42 (1.88 – 6.21)** |  |  |
| Severe | **2.45 (1.39 – 3.96)** | **3.87 (2.56 – 5.86)** | **4.8 (2.84 – 8.11)** | **9 (4.48 – 18.05)** | | **3.44 (1.48 – 7.98)** | **8.3 (4.22 – 16.33)** |  |  |
| Age | 0.99 (0.98 – 1) | **0.98 (0.97 – 0.99)** | 0.98 (0.96 – 1 ) | **0.95 (0.94 – 0.99)** | | 0.98 (0.96 – 1) | **0.97 (0.95 – 0.99)** |  |  |
| Gender |  |  |  |  | |  |  |  |  |
| Man | Ref. |  |  |  | |  |  |  |  |
| Woman | 1.23 (0.91 – 1.65) | **1.51 (1.15 – 1.99)** | **1.57 (1.1 – 2.25)** | 1.12 (0.54 – 1.75) | | 1.11 (0.65 – 1.89) | 1.13 (0.65 – 1.96) |  |  |
| Occupation |  |  |  |  | |  |  |  |  |
| Physician | Ref. |  |  |  | |  |  |  |  |
| Nurse | **1.45 (1.01 – 2.09)** | 1.12 (0.66 – 1.9) | 1.28 (0.65 – 2.5) | 1.06 (0.47 – 2.38) | | 2.48 (0.75 – 8.19) | 1.24 (0.54 – 2.85) |  |  |
| Management | **1.6 (1.07 – 2.39)** | 1.28 (0.73 – 2.27) | 1.16 (0.59 – 2.26) | 0.75 (0.3 – 1.88) | | 1.39 (0.41 – 4.75) | 0.99 ( 0.4 – 2.47 ) |  |  |
| Other | 1.31 (0.83 – 2.06) | 1.07 (0.59 – 1.93) | 1.17 (0.55 – 2.52) | 1.98 (0.81 – 4.82) | | 2.61 (0.74 – 9.29) | 1.63 ( 0.61 – 4.36 ) |  |  |
| Living alone |  |  |  |  | |  |  |  |  |
| No | Ref |  |  |  | |  |  |  |  |
| Yes | 1.23 (0.87 – 1.74) | 0.94 (0.64 – 1.36) | 1.17 (0.78 – 1.76) | 0.67 (0.27 – 1.65) | | 1.15 (0.59 – 2.26) | 1.57 ( 0.94 – 2.62 ) |  |  |

Note: OR=odds ratio, CI=confidence interval, Significant results (p<0.05) are in bold.

Table S2: Associations of Experience of Stigmatization and Discrimination or Violence with Distress and Depression across Years

| Year | Distress | Depressive symptoms |
| --- | --- | --- |
|  | Experience of Stigmatization and Discrimination | |
|  | OR [95% CI] | OR [95% CI] |
| 2020 | 2.93 (2.28-3.76) | 2.46 (1.93-3.13) |
| 2021 | 2.48 (1.96-3.14) | 2.71 (2.11-3.50) |
| 2022 | 1.56 (1.17-2.08) | 1.90 (1.43-2.53) |
|  | Experience of Violence | |
| 2020 | 2.54 (1.53-4.20) | 1.80 (1.08-3.00) |
| 2021 | 2.02 (1.25-3.25) | 1.97 (1.14-3.41) |
| 2022 | 1.72 (1.12-2.64) | 1.41 (1.00-1.99) |

Note: OR=odds ratio, CI=confidence interval.

Table S3: Participant characteristics stratified by number of wave presence

| Variable | Only in one wave | Two or more waves |
| --- | --- | --- |
|  | (N=2516) | (N=1108) |
| Experience of stigmatization or discrimination (n, %) | 616 (24.5%) | 275 (24.8%) |
| Experience of violence (n, %) | **154 (6.1%)** | **38 (3.4%)** |
| Experience of stigmatization, discrimination, or violence (n, %) | 677 (26.9%) | 289 (26.1%) |
| Psychological distress, n (%) | **733 (29.1%)** | **264 (23.8%)** |
| Mild to severe depression, n (%) | **984 (39.1%)** | **378 (34.1%)** |
| Exposure to stressors related to COVID-19, n (%) |  |  |
| None | **676 (26.9%)** | **486 (43.9%)** |
| Mild | **538 (21.4%)** | **185 (16.7%)** |
| Moderate | **511 (20.3%)** | **99 (8.9%)** |
| Severe | **295 (11.7%)** | **65 (5.9%)** |
| Age, mean ± SD | **44.2 (11.9%)** | **45.3 (11.8%)** |
| Women, n (%) | 1819 (72.3%) | 837 (75.5%) |
| Occupation, n (%) |  |  |
| Physician | **804 (32.0%)** | **334 (30.1%)** |
| Nurses | **954 (37.9%)** | **461 (41.6%)** |
| Management | **308 (12.2%)** | **169 (15.3%)** |
| Other | **290 (11.5%)** | **130 (11.7%)** |
| Living alone, n (%) | 339 (13.5%) | 139 (12.5%) |

Note: SD - standard deviation; Differences between waves were assessed using analysis of variance (ANOVA) or chi-squared test. Significant results (p<0.05) are in bold.
